# Analysis of common allergens affecting patients with allergic rhinitis

**DOI:** 10.6026/97320630019024

**Published:** 2023-01-31

**Authors:** Kumari Ranjana, Maheshwari M

**Affiliations:** 1 ENT Department, Madha Medical College and Hospital, Kundrathur Main Road, Kovur, Thandalam, Tamil Nadu - 600122, India; 2 ENT Department, ESI Dispensary, 19, Mandapam Rd, Aspiran Garden Colony, Kilpauk, Chennai, Tamil Nadu 600010, India

**Keywords:** allergic rhinitis, allergen, skin prick test, ENT, respiratory

## Abstract

Allergic rhinitis (AR) is an atopic disorder that affects the quality of life of the patients. AR symptoms include sneezing, nasal congestion, and mucus discharge. It is often associated with several other eye-, ear-, and nose-related symptoms along with fatigue and mood changes. The allergic reaction is triggered by an allergen. An understanding of the allergens that affect a patient is important for allergen avoidance, and ultimately, the treatment of AR. This study aimed to identify the common allergens affecting patients with AR. A total of 52 patients with AR were identified for this study. AR was diagnosed based on the presenting symptoms and measurement of IgE levels and absolute eosinophil counts. Skin prick tests (SPT) were performed to identify the allergen sensitivity of the patients. Patient history, family history, and a detailed account of the symptoms were recorded. Finally, correlation between family history and allergy severity was statistically evaluated. All patients presented symptoms of rhinitis with sinusitis and 61.5% of these were mild or moderate allergic. Few of the patients had ocular or otic symptoms. The duration of allergy was variable in these patients. A high proportion of patients were allergic to house dust mites (92.3%). The proportion of patients allergic to pollen, *Parthenium*, cockroach, cotton dust, and *Aspergillus* were 84.6%, 76.9%, 75%, 65.4%, and 61.5%, respectively. Around 71.2% of patients reported a family history of allergy. SPT severity was not associated with family history (p=0.266). This study successfully identifies the common allergens affecting patients with AR from Chennai, India. It highlights the importance of SPT for the identification of allergens in deciding the treatment regimen for AR.

## Background:

Allergic rhinitis (AR) is an allergic response to innocuous antigens in the environment caused by an inappropriate activation of immune system resulting from excessive production of immunoglobulin (Ig) E [1, 2]. It is the most prevalent atopic disorder that affects around 18% of the worldwide population [3, 4]. And the worldwide prevalence of AR in adults is increasing [4]. In India, the prevalence of AR is 20 to 30% of the population [5]. It affects people of all ages, but 80% of patients are ≤20 years of age [6]. Boys are more affected than girls, but the gender ratio equalizes after puberty [6]. AR adversely affects the quality of life of the patients [3]. It has a negative effect on the physical and mental wellbeing of the patients [2]. Symptoms of AR primarily include sneezing, nasal congestion, and mucus discharge [2]. Patients may also experience fatigue, irritability, as well as mood, cognitive, and sleep disturbance in addition to the nasal, ocular and throat symptoms [7]. AR has important co-morbid associations such as allergic conjunctivitis, chronic sinusitis, serous otitis media, asthma exacerbations, nasal polyposis, sleep apnoea and adenoid hypertrophy [8, 9]. More than 40% of patients with AR report fatigue in spite of good sleep [10]. Exposure to allergens, like different varieties of dust mites or moulds, can happen via skin contact, injection, ingestion, or inhalation. Approximately 10,000 L of air laden with aero-allergens passes over the mucosa per day. The trapped allergens elute and diffuse into the mucosal layer. The allergens are then processed by antigen-presenting cells (APCs) and presented to the helper T cells (Th) 2, which in turn secrete cytokines including interleukin (IL)-4 and IL-13 [8,9]. These cytokines induce B cells to synthesize IgE that bind to Ig receptors on mast cells [9]. Re-exposure to the allergen activates the mast cells to release histamines [8]. Early phase response constitutes release of leukotrienes - (LT)C4, LTD4, and LTE4 - and prostaglandin D2 that lasts for 2 to 3 h [8]. This is accompanied by sneezing rhinorrhoea and nasal congestion [8]. The late phase response initiates after 4 to 6 h and persists for 18 to 24 h [8]. The mucosa gets infiltrated by eosinophils, neutrophils, basophils, T cells and macrophages [6]. These cells further release mediators that cause inflammation and continuation of nasal symptoms [6,7,8, 9]. Several different cytokines up regulates adhesion molecules like vascular cell adhesion molecule (VCAM)-1 [8]. VCAM-1 on blood vessel walls interacts with VLA-4 receptor on cells and recruits peripheral blood cells (eosinophils and basophils) to the target inflammatory site causing chronicity [11]. This study aimed to identify and analyze the common allergens affecting patients with AR within Chennai, a cosmopolitan city in India. We have also studied the presentation of symptoms and family history of the patients.

## Materials and Methods:

## Study population:

A total of 52 patients were identified in this study. All patients presenting to the ear, nose, throat (ENT) outpatient department (OPD) with allergic symptoms were included in this study. Patients with other comorbidities were excluded. Informed consent was taken from the patients before the study. This study was retrospectively approved by an independent ethics committee. This study was conducted in Madha Medical College, Chennai, India from 2018 to 2022.

## Study procedure:

Patients were subjected to a thorough general and ENT exam, skin prick test (SPT) for allergy, and blood tests for measuring IgE level and absolute eosinophil count. The IgE levels and absolute eosinophil counts were done for diagnostic purposes. Patient history suggestive of allergy was documented during the clinical examination. Presence or absence of certain symptoms and allergy duration were also documented.

## Skin prick test:

SPT was performed as described by Latha et al. 2011 [12]. Briefly, allergen extract was injected intradermally, and the site of injection was examined after 15 minutes for signs of an allergic reaction. The allergens tested included house dust mites, cotton dust, *Aspergillus* extract, pollen from trees, *Parthenium* pollen, cockroach extract, and cat dander and dog fur. SPT reactivity was graded as no allergy, mild allergy, moderate allergy, and severe allergy based on area of induration.

##  Statistics:

The results from the family history and allergy severity were tabulated and statistically analyzed for correlation using Chi squared test. SPSS version 17 was used for the analysis.

## Results

## Patient demographics

All 52 patients identified in this study were Asian and presented symptoms of rhinitis with sinusitis (Table 1). The minimum age ofthe patients was 10 years and maximum age was 66 years. All patients were diagnosed with AR. A high proportion of the patients were mild or moderate allergic (61.5%).

## Allergy assessment

Presentation of allergic symptoms of the patients were further categorized - nasal congestion, cough, sneeze, headache, wheezing, cold, running nose, reddish eye, ear pain, itching, watery eyes, rashes, and mouth breathing. Most of the patients had sneezing, followed by nasal congestion, cold, running nose, and headache (Table 2). Very few patients complained itching, wheezing, ear pain, watery eyes, rashes, and reddish eye. Only one patient complained of mouth breathing. Of the 12 (25.5%) patients with itching, 1 (2.1%) patient, 2 (4.3%) patients, and 9 (19.1%) patients had itching in the palms, soles, and eyes, respectively. Of the 12 (23.6%) patients with wheezing, 6 (11.8%) patients, 5 (9.8%) patients, and 1 (2%) patient had mild, moderate, and severe wheezing, respectively. The duration of presentation of allergic symptoms was variable in the patients assessed in this study (Table 3).

## Allergen assessment:

Patients were tested against specific allergens using SPT (Table 4). Most patients were allergic to house dust mites (92.3%). The proportion of patients allergic to house dust mites was the highest, followed by pollen (84.6%), *Parthenium* (76.9%), cockroach (75%), cotton dust (65.4%), and *Aspergillus* (61.5%). The patients tested in this study were least affected by cat dander (1.9%) and dog fur (1.9%).

## Family history

We also asked the patients with AR about any family history of allergy. While 71.2% of patients reported a family history of allergy, relatively lower proportion of patients reported allergy on either their paternal or maternal side of the family (Table 5).

##  Association of family history with AR severity:

Next, we analyzed if AR was associated with a family history of allergy. In the patients analyzed in this study, SPT severity was not associated with family history (p=0.266). There was no significant association between wheezing and SPT severity (p>0.05), and allergy duration and SPT severity (p>0.05).

## Discussion:

This study assessed 52 patients with AR for their symptoms, common allergens, severity of allergy, and association with family history. House dust mite was the most common allergen followed by pollen, *Parthenium*, cockroach, cotton dust, *Aspergillus*, cat dander, and dog fur. Thirty seven (71.2%) patients reported a family history of allergy. However, family history was not significantly associated with severity of the allergic reaction in the SPT. We had published a similar study previously and this study conforms to the previous data [13]. House dust mite is a common allergen affecting AR symptoms. And family history is not a predictor of severity. In our previous study, many patients were allergic to dog fur and cat fur [13]. However, in this study only 2 patients each were allergic to dog fur and cat dander. Patients with AR susceptible to house dust mites are at a risk of developing asthma over time [14]. In a study from Bangalore, another cosmopolitan city in India, 41% of the patients were allergic to house dust mites as tested by SPT [15, 16]. House dust mite was the foremost allergen in patients with allergic airway disease from South-western Maharashtra, a region from India [16]. It is a common allergen affecting several patients with AR from different parts of the world including India, Hong Kong, China, Ireland, Qatar, and Belgium [17,18,19,20, 21,22,23]. It has been reported as the most common allergen for AR [14]. Our study results corroborate these previous findings. Reaction to an allergen in SPT is not a surrogate for disease severity [9]. And this study does not associate family history with disease severity. An intradermal test or SPT is an in vivo test used to identify the allergen and to show an IgE-mediated sensitization [24]. Upon activation by an allergen, skin induration is observed which can be graded [24]. SPT is sensitive and specific and is recommended by several international guidelines [25, 26]. We have used the recommended SPT to identify the common allergens in this study. Though multiple family members are usually affected in AR, specific sensitivities do not appear to be simple heritable traits [8]. The factors affecting the severity of AR are likely to be multifactorial [27]. Environmental factors, lifestyle, socioeconomic status, air pollution, exposure to new allergens, and stress are associated with the incidence of AR [27]. In this study, genetic factors were not associated with disease severity. But, they may be associated with the incidence of AR, which was not assessed in this study. The combination of IgE levels and medical history of the patient is considered “the gold standard” in defining AR in clinical practice [4]. We measured IgE levels and absolute eosinophil counts for diagnosing AR. We also recorded the presentation of symptoms in detail. However, we do not categorize AR as seasonal or perennial. But we do present the duration of allergic symptoms, which indicates (but not confirms) an equal distribution of patients with seasonal AR and perennial AR in this study population. We did not test the correlation of IgE levels and absolute eosinophil counts with disease severity. We also did not assess the effect of the disease on the quality of life of the patients. AR affects the quality of life of the patients [7,8,9, 10]. Allergen avoidance is important for preventing AR [9]. In order to avoid the allergens, they need to be identified. We have identified the common allergens that affect the patients in this study and allergen avoidance was a part of their treatment regimen.

## Conclusion:

This study successfully identifies the common allergens affecting the patients with AR. It also highlights the importance of SPT and identification of allergen sensitivity in the treatment of AR.

## Figures and Tables

**Table 1:** Patient demographics

Characteristics	n=52
Females n (%)	29 (55.8)
Age mean (SD)	31.54 (13.323)
Allergy severity n (%)	
Mild/moderate allergic	32 (61.5)
Severe allergic	20 (38.5)
N - Number of patients; SD - standard deviation

**Table 2:** Presentation of symptoms

Presentation of symptoms	Proportion of patients n (%)
Nasal congestion (n=52)	27 (51.9)
Cough (n=52)	13 (25)
Sneezing (n=52)	37 (71.2)
Headache (n=43)	19 (44.2)
Wheezing (n=51)	12 (23.6)
Cold (n=52)	25 (48.1)
Running nose (n=52)	23 (44.2)
Reddish eye (n=52)	2 (3.8)
Ear pain (n=52)	8 (15.4)
Itching (n=47)	12 (25.5)
Watery eyes (n=42)	3 (7.1)
Rashes (n=28)	2 (7.1)
Mouth breathing (n=26)	1 (3.8)

**Table 3:** Allergy duration

Allergy duration	Proportion of patients n (%)
6 weeks	5 (9.6)
6 weeks to 6 months	9 (17.3)
6 months to 1 year	5 (9.6)
1 to 5 years	7 (13.5)
5 to 10 years	6 (11.5)
10 years	2 (3.8)
N - Number of patients

**Table 4:** Identification of common allergens and severity assessment

Allergen	No allergy n (%)	Mild allergy n (%)	Moderate allergy n (%)	Severe allergy n (%)
House dust mites	4 (7.7)	12 (23.1)	19 (36.5)	17 (32.7)
Cotton dust	18 (34.6)	23 (44.2)	10 (19.2)	1 (1.9)
*Aspergillus*	20 (38.5)	19 (36.5)	13 (25)	0 (0)
Pollen	8 (15.4)	22 (42.3)	21 (40.4)	1 (1.9)
*Parthenium*	12 (23.1)	15 (28.8)	24 (46.2)	1 (1.9)
Cockroach	13 (25)	9 (17.3)	25 (48.1)	5 (9.6)
Cat dander	50 (96.2)	0 (0)	1 (1.9)	1 (1.9)
Dog fur	50 (96.2)	0 (0)	2 (3.8)	0 (0)
N, number of patients

**Table 5:** Family history of allergy

Category	Proportion of patient's n (%)
Family history	37 (71.2)
Paternal history	14 (26.9)
Maternal history	15 (28.8)
N, number of patients
